# FlyAtlas 2 in 2022: enhancements to the *Drosophila melanogaster* expression atlas

**DOI:** 10.1093/nar/gkab971

**Published:** 2021-10-29

**Authors:** Sue A Krause, Gayle Overend, Julian A T Dow, David P Leader

**Affiliations:** Institute of Molecular, Cell & Systems Biology, College of Medical, Veterinary & Life Sciences, University of Glasgow, Glasgow G12 8QQ, UK; Institute of Molecular, Cell & Systems Biology, College of Medical, Veterinary & Life Sciences, University of Glasgow, Glasgow G12 8QQ, UK; Institute of Molecular, Cell & Systems Biology, College of Medical, Veterinary & Life Sciences, University of Glasgow, Glasgow G12 8QQ, UK; Institute of Molecular, Cell & Systems Biology, College of Medical, Veterinary & Life Sciences, University of Glasgow, Glasgow G12 8QQ, UK

## Abstract

FlyAtlas 2 (flyatlas2.org) is a database and web application for studying the expression of the genes of *Drosophila melanogaster* in different tissues of adults and larvae. It is based on RNA-Seq data, and incorporates both genes encoding proteins and microRNAs. We have now completed the population of the database with 13 tissues from both male and female adults, five sex-specific tissues, and eight larval tissues. Larval garland cell nephrocytes have also been included. Major enhancements have been made to the application. First, a facility has been added for a ‘Profile’ search for genes with a similar pattern of tissue expression as a query gene. This may help establish the function of genes for which this is currently unknown. Second, a facility has been added dedicated to the larval midgut, where the difference in gene expression in the five regions of different pH can be explored. A variety of further improvements to the interface are described.

## INTRODUCTION

The FlyAtlas database was established in 2007, providing information on the expression of different genes in a wide range of tissues of one of the most important model organisms, *Drosophila melanogaster* (1). The original database was constructed from microarray hybridization data, continues to serve the scientific community (2) and is now integrated in the FlyBase informatic framework (3). After the technique of RNA-Seq (4) was introduced, a new version of FlyAtlas was constructed in 2018 using this methodology. This database and associated web application is FlyAtlas 2 (5), and includes information on both mRNA and microRNA transcripts. It also provides separate information for the tissues of male and female adult flies.

The initial version of FlyAtlas 2 had three facilities, allowing individual searches on the basis of gene identifier, searches for multiple genes satisfying a particular verbal description or gene ontology, and searches for the most expressed genes in specific tissues. We have now added a further facility where the search criterion is the pattern of expression of a query gene across different tissues, allowing genes of similar expression profile to be identified, indicating a potential shared function with the query.

One focus of research in our own laboratory has been the *Drosophila* alimentary canal, because of its close functional analogies with the alimentary and renal system of mammals, and its critical roles in insect metabolism and homeostasis. Components of this—the midgut, hindgut, and Malpighian tubules—are represented among the tissues in FlyAtlas 2. One interesting feature of the *Drosophila* larval midgut is that it is composed of five regions of widely different pH: the neutral gastric caeca/anterior region, the acidic region, the neutral region, the slightly acidic transitional region, and the posterior alkaline region (6). These map closely to domains documented in larval and adult midguts based on other criteria (6,7), increasing confidence in their functional significance. As part of previous studies of transcripts in the acidic region of the midgut (8), we prepared an RNA-Seq library from all five regions of the larval midgut. We have now made these data generally available and searchable in a new facility within FlyAtlas 2.

## DATA COLLECTION AND TECHNICAL IMPLEMENTATION

Details of rearing *Drosophila melanogaster*, dissection of whole tissues and preparation of RNA for sequencing are described in the original report of FlyAtlas 2 (5). The larval garland cell nephrocytes were prepared by collecting feeding third instar Canton S larvae. The dissection involved removing the bottom half of the larval body, cutting the midgut. The cuticle of the top half was gently moved away to expose the proventriculus of the midgut, and while holding onto the midgut, the garland cells were gently removed. The garland cells for individual triplicates were pooled and transferred to a tube containing Qiazol, and total RNA was isolated as previously described.

**Figure 1.**
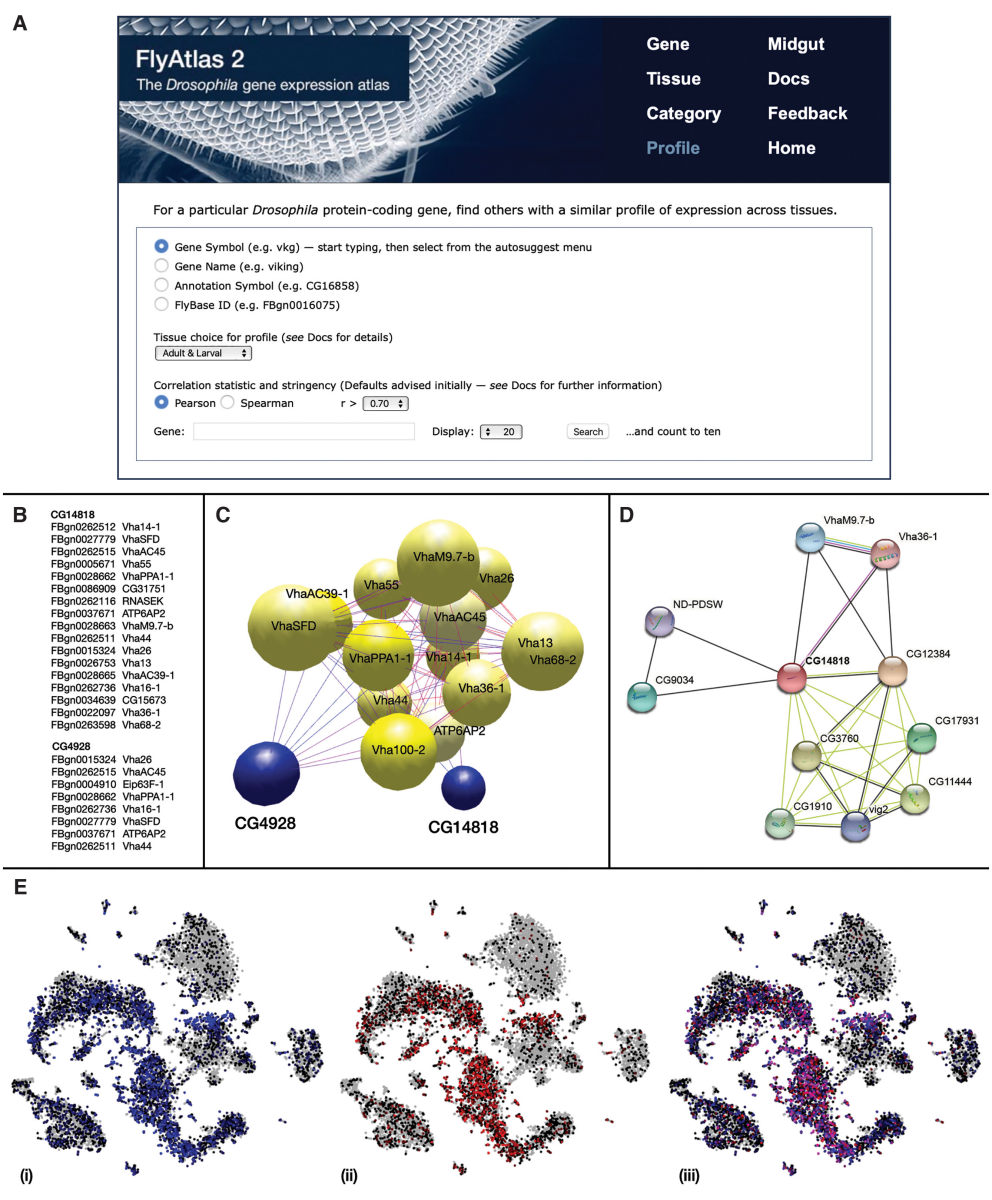
Profile search. (**A**) Part of web interface, showing user options. See Supplementary Figure S1 for example of results display. (**B**) Listing of genes with tissue expression profiles resembling *CG4928* and *CG14818* at Pearson correlation *r* ≥ 0.70 and Bonferroni-corrected probability *P*_B_≤ 0.05, in descending order of *r*. (**C**) Three-dimensional clustering of *CG4928*, *CG14818* and *vha* genes (yellow spheres) after correlation analysis with *BioLayout*. (**D**) STRING cluster of the uncharacterized gene, *CG14818*, showing connection to *Vha36-1* and *VhaM9.7-b*. The colouring of the edges (lines) reflects the status of the interactions: purple/red—known from experimental determination, blue—known from curated databases, light green—predicted from gene neighbourhood, and black—co-expression. (**E**) SCope representation of the single cell data for adult tubules from the Fly Cell Atlas consortium. (i) *Vha36-1* (blue), (ii) *CG14818* (red), (iii) *Vha36-1* and *CG14818*.

For the regions of the midgut, details are as follows. Mid-L3 Canton S larvae were maintained on 0.1% bromocresol purple *Drosophila* diet for 2 hours, and their midgut excised. Each midgut was dissected into the five regions of pH shown in Figure 1 of (8), which were separately stored in RLT buffer (Qiagen) as triplicate pooled samples. Whole RNA was extracted using the RNAeasy Mini Kit (Qiagen), and quality/quantity determined on an Agilent 2100 Bioanalyzer. Samples were run on an Illumina Genome Analyzer 11x by the Glasgow University Polyomics Facility, and single-end reads generated. It should be noted that microRNA libraries were not prepared for the midgut regions.

Technical details of the database and web application are generally as described previously (5). At the time of writing the database had been updated from FlyBase files, fbgn_annotation_ID_fb_2021_04.tsv and gene_snapshots_fb_2021_04.tsv, for gene nomenclature, and gene_association_v2.1.fb (2021-08-04) and go-basic.obo (2021-09-01) for gene ontology (9).

The additional ‘Profile’ search used the Apache commons-math-2.2 java packages (https://commons.apache.org/proper/commons-math/) to calculate Pearson and Spearman correlation coefficients and the Bonferroni-corrected probability. The scalable vector graphics images for the results of queries to the Midgut facility were generated with version 1.7 of the Apache™ Batik package (xmlgraphics.apache.org/batik/). The web interface was originally designed to be ‘responsive’ to mobile input devices, and this feature has been maintained in the new facilities.

## NEW FEATURES

### Profile search

The Profile search is a facility that takes a query gene and compares the pattern of expression among different tissues to other genes in the database, returning those with a correlation coefficient (*r*) greater than a particular cut-off value. The user can choose between Pearson and Spearman correlation coefficients, and vary the cut-off value. However defaults have been chosen to provide a reasonable starting point in most cases (Figure 1A). By default, FPKM values (Fragments Per Kilobase of transcript per Million mapped reads) from 21 different adult and larval tissues are used to generate a ‘profile’, with only one sex being chosen for adult tissues to avoid bias. Because of computer memory limitations certain tissues are excluded from this default, but the user has the option of selecting alternative profiles that include only adult, adult male, adult female, larval or intestinal tissues (Supplementary Table S1).

It is important to emphasize the difference between the Profile search in FlyAtlas 2 and a similarly named facility on FlyBase that uses data from the modENCODE project (flybase.org/rnaseq/profile_search). In the latter facility one specifies particular expression criteria and the search finds corresponding genes, whereas in the one described here one specifies a particular gene of interest and the search retrieves other genes with a similar pattern of expression across tissues.

An illustration of how the Profile search can be combined with other approaches to investigate genes of unknown function is provided by studies with two such genes, *CG4928* (FBgn0027556) and *CG14818* (FBgn0026088). These genes were identified in Profile searches of members of the vacuolar H^+^-ATPase (*vha*) family, and found to have similar patterns of tissue expression to some members of this family (Figure 1B). Inspection of the tissue expression data made it clear that the relationship was imperfect: *CG4928* is expressed most in Malpighian tubules whereas the *vha* family is expressed most in hindgut, and *CG14818* is more weakly expressed than the *vha* family. However, we decided to perform more extensive correlation analysis using the software *BioLayout* (10) comparing the expression pattern of each gene with all others in a set of 14 related *vha* genes and the two of ill-defined genes. The results can be visualized as three-dimensional interactions between spheres, and are shown in Figure 1C, where it can be seen that *CG4928* and *CG14818* are still associated with the *vha* cluster after this more rigorous analysis, although they lie at different points on its periphery. The value of such analyses of correlated expression of genes in different tissues is illustrated by showing how it can be combined with other approaches. We applied the protein–protein interaction network resource STRING (11) to these two genes and found that *CG14818* (but not *CG4928*) was physically associated with the products of two genes of the *vha* family—*Vha36-1* and *VhaM9.7b*—from the original profile search (Figure 1D). Further analysis was performed using the SCope tool (12,13), which maps *Drosophila* genes to specific cell types. Figure 1E shows the expression patterns of the transcripts for *CG14818* and *Vha36-1* in the adult tubule (13) are very similar, although not identical.

### Larval midgut facility

**Figure 2.**
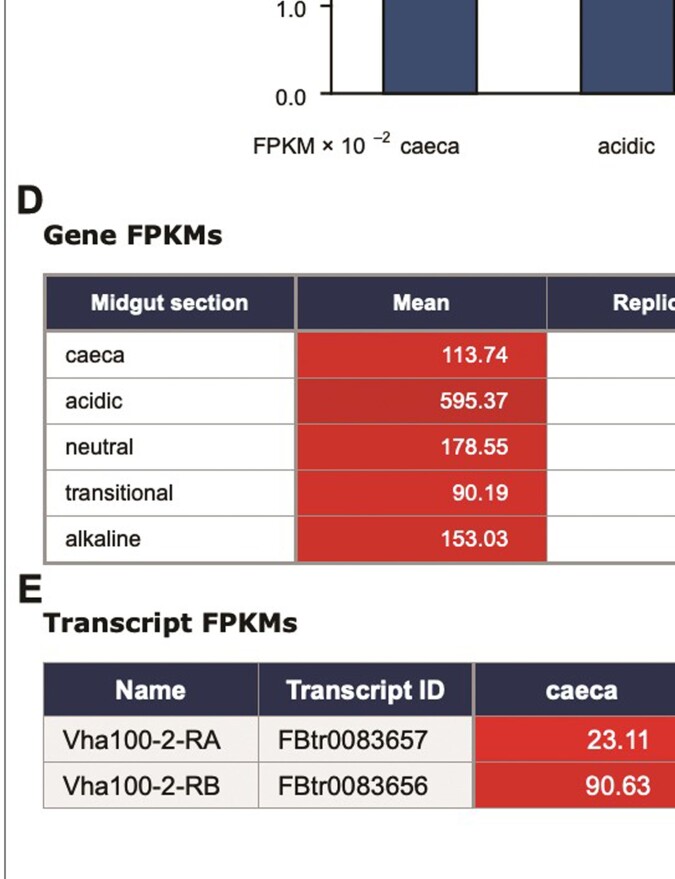
Interface to the larval midgut facility and presentation of results. (**A**) Illustration of the different regions of the midgut represented by the data. (**B**) Entry options for gene identifier (as for standard Gene search). (**C**) Graphical presentation of gene FPKMs for different regions (not available in standard Gene search for space reasons). (**D**) Tabular presentation of gene FPKMs with individual replicate values (not in standard Gene search) and standard deviations. The background colour intensity increases with magnitude. (**E**) Tabular presentation of transcript FPKMs. It can be seen that in this example, although the major transcript Vha100-2-RB is most abundant in the acidic region, the minor transcript Vha100-2-RA is most abundant in the alkaline region.

Larval tissues were chosen in previous work on different regions of midgut, both to complement an existing online resource for adult tissue (flygut.epfl.ch/) and because the main criterion was functional (gut pH) rather than morphological (8). It was decided to provide a separate interface for this within FlyAtlas 2 rather than incorporate it into the standard search by gene identifier, partly to avoid cluttering the latter with extra information of specialist interest and partly because of the complication caused by the lack of microRNA data for the midgut. The dedicated interface allowed clearer presentation of results, with a graphic indicating the different anatomical regions (Figure 2). An example of the distinct patterns of gene expression that can occur in these different regions of the larval midgut is illustrated by results with five different serine proteases (Supplementary Figure S2A). Comparison with the results of a study of roughly analogous regions of the adult midgut (6) show both similarities and differences (Supplementary Figure S2B) between the adult and larval midgut regions.

### Other enhancements

There have been several routine improvements since our original report of FlyAtlas 2. The outstanding data for accessory glands, crop, eye, heart, thoracoabdominal ganglia, rectal pad and virgin and mated spermatheca have now been added. We have included links out from genes to the recent related metabolomic database ‘FlyMet’ (flymet.org) and, where appropriate, the neuropeptide database ‘DINeR’ (14). We have also tried to synchronize the gene descriptors and ontologies with those at FlyBase (3) as they are updated. Enhancements that deserve specific mention are:

#### Inclusion of garland cells

**Figure 3.**
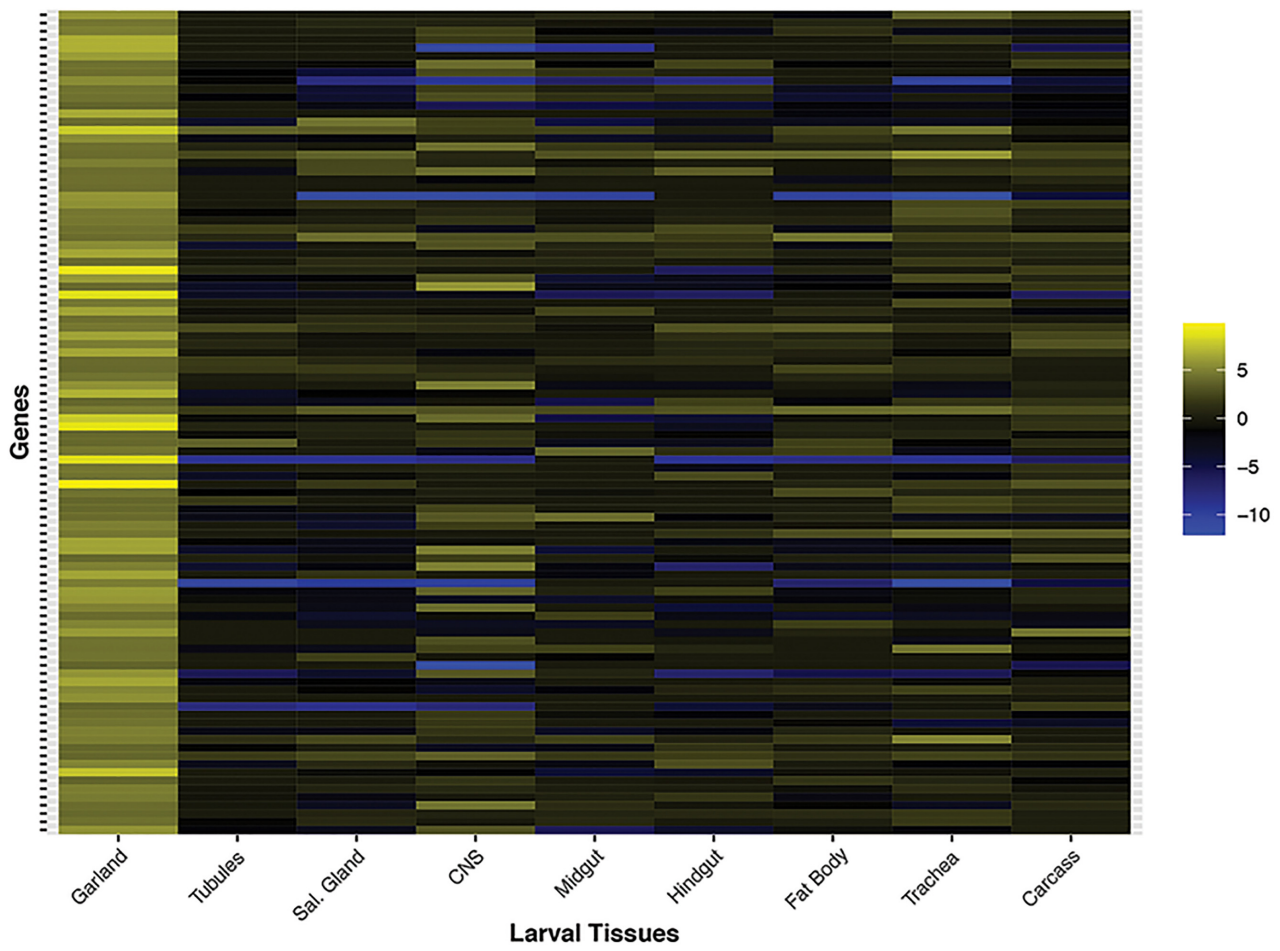
Unique nature of the larval garland cell transcriptome. A heatmap of larval tissues focusing on the top 100 most expressed genes (excluding ribosomal protein genes) is shown. The RNA-Seq data for the main larval tissues were analysed for the log_2_ fold change of their FPKM in comparison to the whole larval transcriptome.

The garland cell nephrocytes of *Drosophila* larvae are of particular interest because they share a slit diaphragm structure with human podocytes (15), and provide a genetically manipulable model of human nephrotic syndrome (16). Including them in FlyAtlas 2 increases the utility of the resource for studying insect models of human kidney disease. Figure 3 shows that there is very little overlap between the larval tubule and the garland cell transcriptomes. The most highly enriched nephrocyte genes in FlyAtlas 2 include well-known participants in slit diaphragm function, such as *amnionless* (17), but also otherwise undocumented genes such as *CG34296*, which appears to be nephrocyte-specific.

#### Category search by FlyBase groups

The Category facility of FlyAtlas 2 allows one to search for genes of a particular functional type on the basis of terms or identifiers from FlyBase gene ontology tables. Nevertheless, this does require that one's term of interest exists in these tables. More recently FlyBase undertook a classification of *Drosophila* genes as members of functional groups (18), which suggested the possibility of providing users with a drop-down list of groups to select from, in addition to being able to search by entering a term of their choosing. In practice, implementation was complicated by the number of groups, their hierarchical structure, and the fact that some groups have very many members. We have therefore restricted the list to 126 particular groups with from 3 to 260 members (Supplementary Table S2), covering 3731 genes in total. The interface is shown in Supplementary Figure S3. This provides the user with another option for Category searches, and although the choice of groups is somewhat subjective, it will be kept under review and revised if necessary.

#### Batch input

Some users had requested an option to input a list of genes to FlyAtlas 2, rather than search for each individually. Although APIs are provided for programmatic access to the database, their use requires informatics expertise that is not available to all users. We have now added an option in the Gene search facility for batch input (Supplementary Figure S4). As well as dealing with this problem it allows users to examine custom sets of genes together.

## CONCLUSION

With the advent of single-cell transcriptomics, providing data on both whole organisms (13) and individual tissues (7,19,20), it is worth emphasising some of the reasons why tissue transcriptomic atlases continue to be useful. For example, it has been demonstrated that whole-organism transcriptomics can severely under-represent tissue-specific expression (1). Moreover, single-cell RNA-Seq requires much more amplification by PCR than bulk RNA-Seq or microarray technologies, so that FlyAtlas and FlyAtlas 2 can be expected to report lower abundance transcripts more accurately. In addition, FlyAtlas and FlyAtlas 2 (and the metabolomic resource FlyMet.org) were systematically produced from the same fly lines under the same conditions in the same laboratory, and so possess a desirable comparability. Finally, FlyAtlas 2 explicitly sequenced not just male and female tissues separately, but also sequenced and curated microRNA species, some of which show extreme levels of tissue specificity. Therefore, although single cell transcriptomics offer a major advance in our understanding of how cells co-operate to make functioning tissues, tissue-level transcriptomes provide sensitive data to complement and corroborate the new technology.

This point of view is borne out be the fact that the web application continues to attract over 31 000 unique page views per year, and the article describing it has been cited over 160 times, according to Google Scholar. We expect that the new features that have been added will enable FlyAtlas 2 to be even more useful to the scientific community in the future.

## DATA AVAILABILITY

The FlyAtlas 2 web application is freely accessible to all without registration. A MySQL ‘dump’ of the FlyAtlas 2 database can be downloaded from the ‘Documentation’ page of flyatlas2.org. RNA-Seq data have been deposited with European Nucleotide Archive under accession numbers PRJEB22205 and PRJEB11865, and are publicly available.

## Supplementary Material

gkab971_Supplemental_File
