# Supplementary material for: FlyAtlas 2 in 2022: enhancements to the Drosophila melanogaster expression atlas
Source: Nucleic Acids Res. 2021 Oct 29;50(D1):D1010–5. doi: 10.1093/nar/gkab971 (PMC8728208; doi:10.1093/nar/gkab971)
Supplement: gkab971_Supplemental_File [file gkab971_supplemental_file.pdf]

Supplementary Materials

FlyAtlas 2 in 2022: enhancements to the *Drosophila melanogaster* expression atlas

Sue A. Krause, Gayle Overend, Julian A.T. Dow and David P. Leader

Institute of Molecular Cell and Systems Biology, College of Medical, Veterinary and Life Sciences, University of Glasgow, Glasgow G12 8QQ, UK

Table of Contents

Figure S1. Presentation of results from Profile search 2
Figure S2. Expression of genes in different regions of midgut 3
Figure S3. Interface to Category search 5
Figure S4. Interface to Gene batch search 6
Table S1. Available tissue selections for Profile search 7
Table S2. FlyBase groups employed in Category search 8

Figure S1 Presentation of results from a Profile search

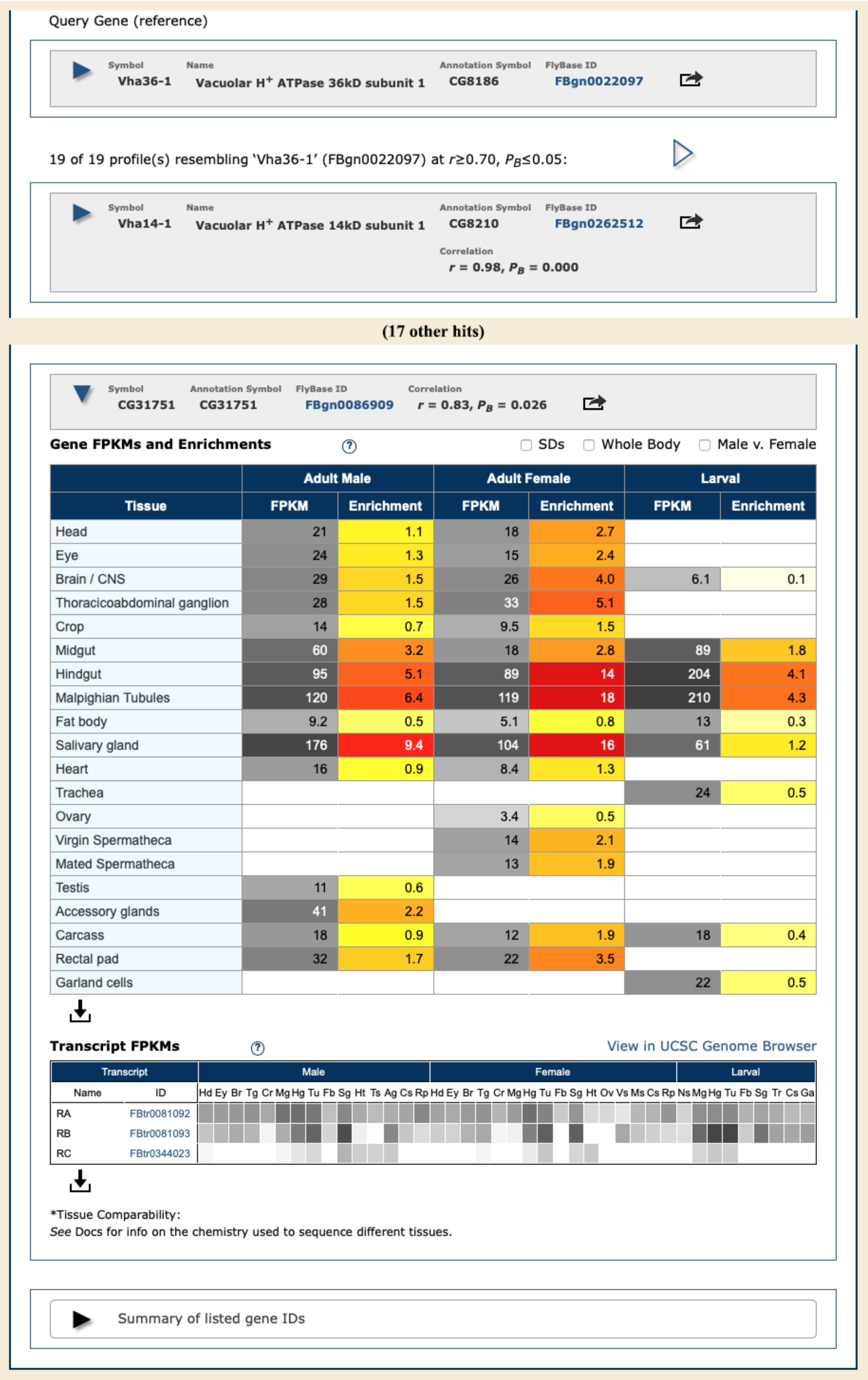

Query Gene (reference)

Symbol	Name	Annotation Symbol	FlyBase ID
Vha36-1	Vacuolar H$^+$ ATPase 36kD subunit 1	CG8186	FBgn0022097

19 of 19 profile(s) resembling 'Vha36-1' (FBgn0022097) at r≥0.70, P_B≤0.05:

Symbol	Name	Annotation Symbol	FlyBase ID
Vha14-1	Vacuolar H$^+$ ATPase 14kD subunit 1	CG8210	FBgn0262512

Correlation
r = 0.98, P_B = 0.000

(17 other hits)

Symbol	Annotation Symbol	FlyBase ID	Correlation
CG31751	CG31751	FBgn0086909	r = 0.83, P_B = 0.026

Gene FPKMs and Enrichments ? ☐ SDs ☐ Whole Body ☐ Male v. Female

Tissue	Adult Male FPKM	Adult Male Enrichment	Adult Female FPKM	Adult Female Enrichment	Larval FPKM	Larval Enrichment
Head	21	1.1	18	2.7		
Eye	24	1.3	15	2.4		
Brain / CNS	29	1.5	26	4.0	6.1	0.1
Thoracicoabdominal ganglion	28	1.5	33	5.1		
Crop	14	0.7	9.5	1.5		
Midgut	60	3.2	18	2.8	89	1.8
Hindgut	95	5.1	89	14	204	4.1
Malpighian Tubules	120	6.4	119	18	210	4.3
Fat body	9.2	0.5	5.1	0.8	13	0.3
Salivary gland	176	9.4	104	16	61	1.2
Heart	16	0.9	8.4	1.3		
Trachea					24	0.5
Ovary			3.4	0.5		
Virgin Spermatheca			14	2.1		
Mated Spermatheca			13	1.9		
Testis	11	0.6				
Accessory glands	41	2.2				
Carcass	18	0.9	12	1.9	18	0.4
Rectal pad	32	1.7	22	3.5		
Garland cells					22	0.5

The output from an example Profile search with gene *Vha36-1* is shown. Results for each hit are initially shown in summary form (e.g. *Vha14-1*), which can be expanded by the triangles to present the actual expression data (*CG31751*).

Figure S2 Expression of genes in different regions of the midgut

A

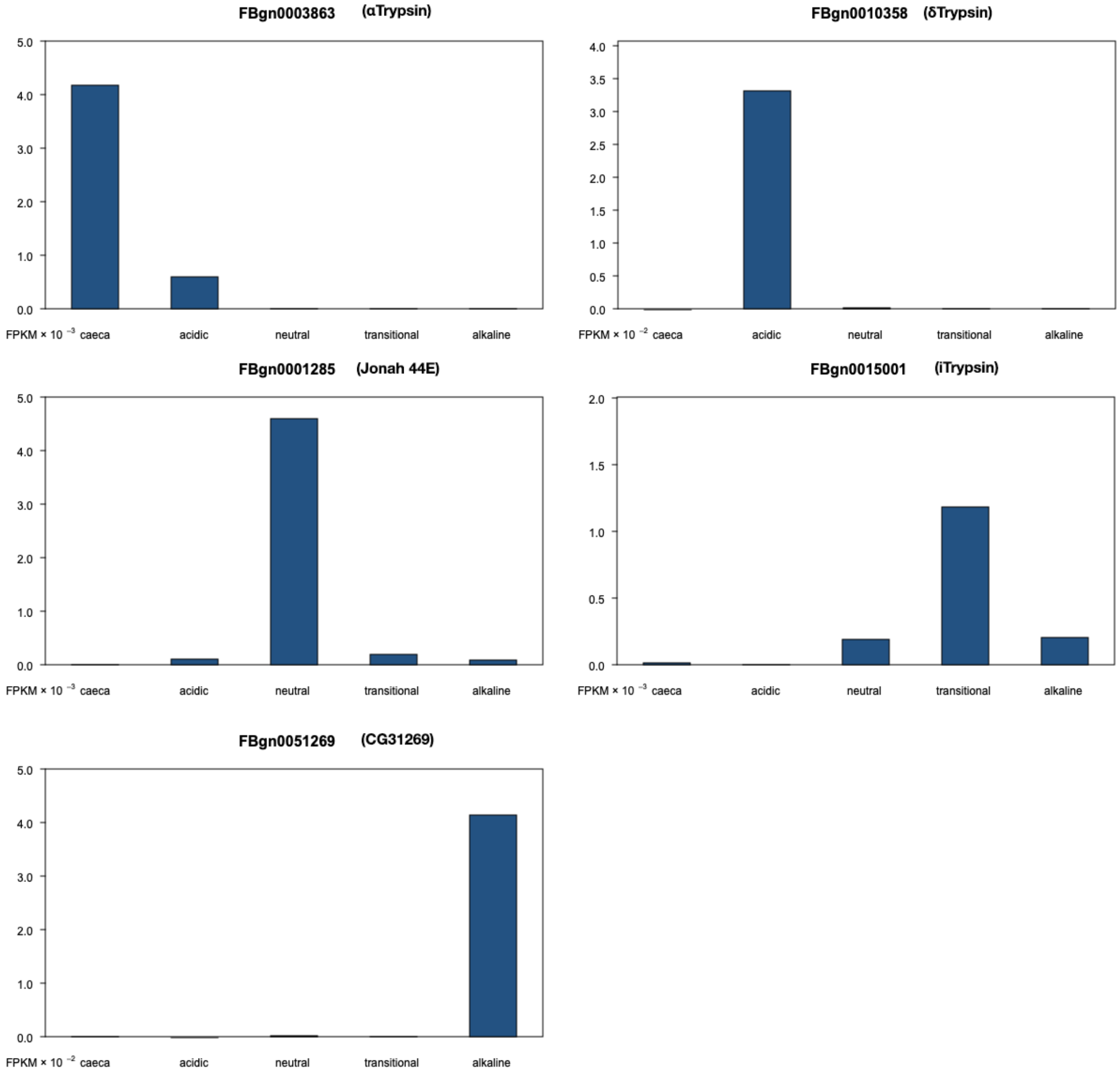

Region-specificity of expression of certain genes in the different pH regions of larval midgut illustrated by selected serine-proteases.

Figure S2 cont.

B

Gene	Adult Midgut	Larval Midgut
alphaTry (CG1844)	R2	caeca
LpR1 (CG31094)	R2	caeca, acidic
CG8690	R2	caeca, acidic
CG8177	R3	acidic
Ptx1 (CG1447)	R3	acidic
Lab (CG1264)	R3	acidic
PGRP-LB	R3, R4	acidic, neutral (caeca, transitional)
PGRP-SC1	R4	(acidic) neutral
CG2772	R4	neutral, transitional
CDase	R4	neutral, transitional
mag	R4	caeca, (acidic), neutral
CG17145	R4	caeca
cry	R4	caeca
Chymotrypsin (CG31267)	R5	alkaline
caudal (CG1759)	R5	neutral, transitional, alkaline

Comparison of expression of genes in single regions of larval midgut (FlyAtlas 2 analysis) and adult midgut (Buchon *et al.* 2013 Cell Rep, 3, 1725-1738). There is obviously no exact correspondence between the four regions — R2 to R4 — of the adult and the five regions — caeca, acidic, neutral, transitional and alkaline, of the larva. Nevertheless, it is possible to identify similarities (highlighted in green) and differences (highlighted in salmon).

Figure S3 Interface to Category search

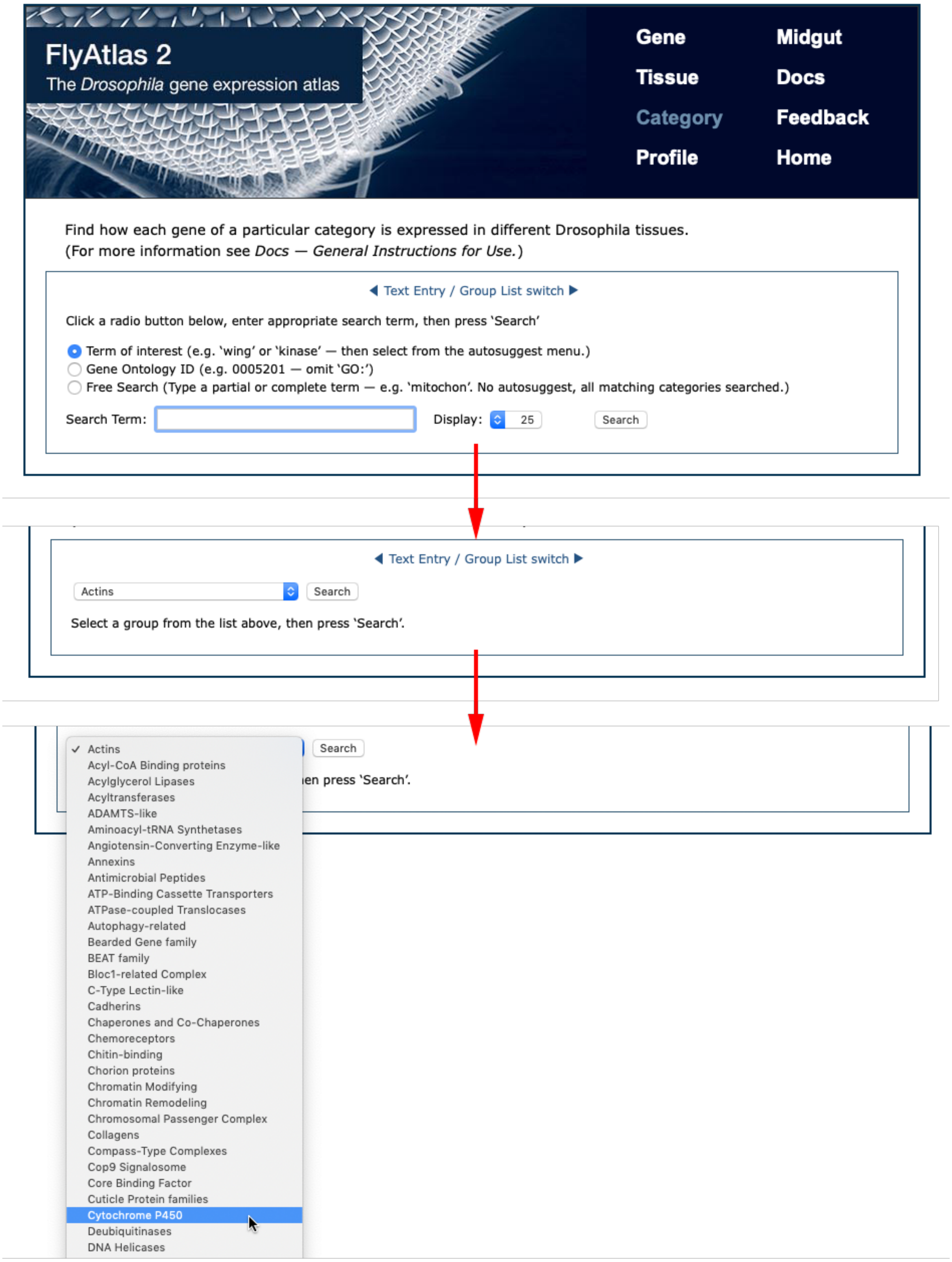

The initial search form is shown for entry of a single term, the alternative for selecting a FlyBase group, and a section of the pull-down list showing some of the options available.

Figure S4 Interface to Gene batch search

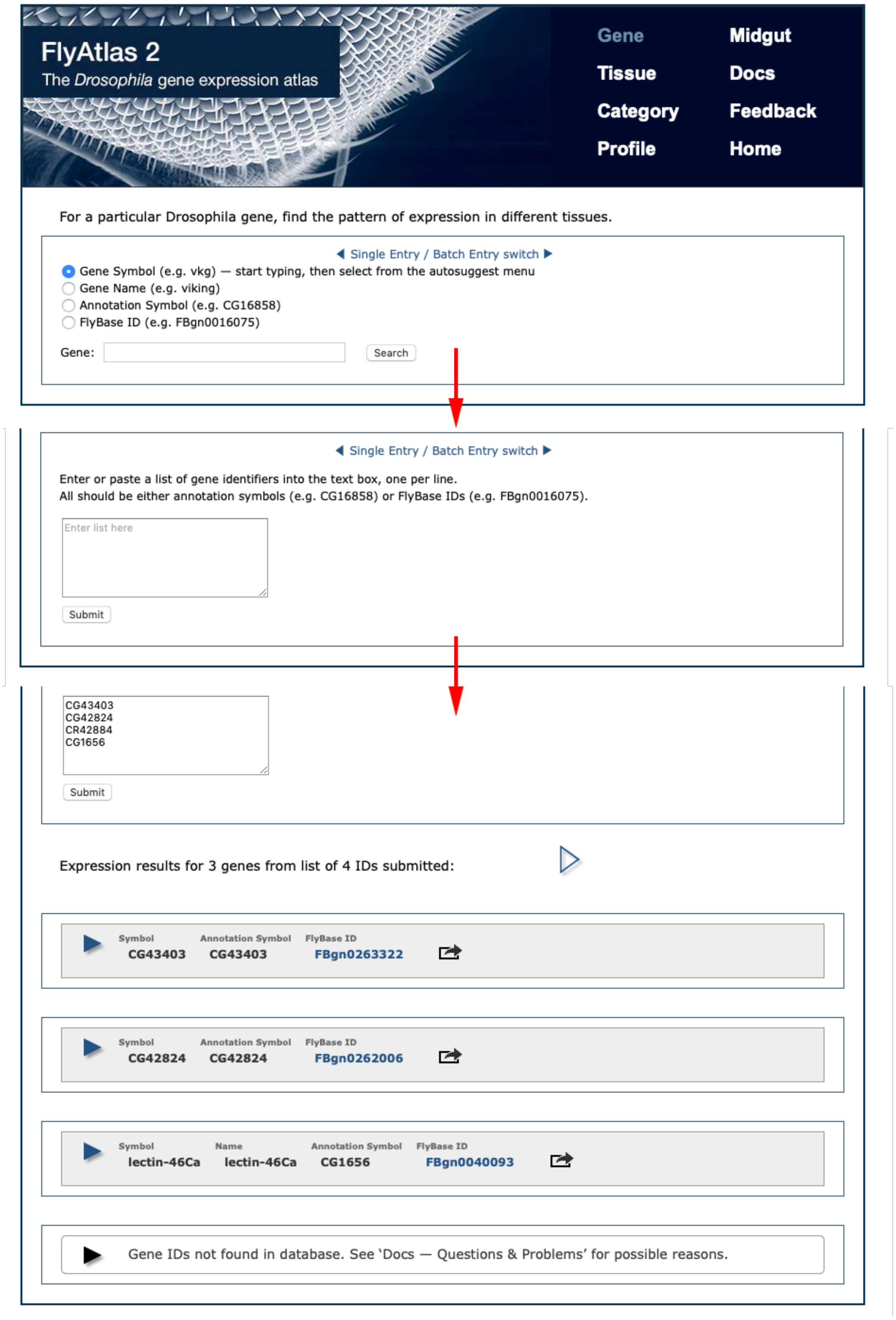

The initial search form is shown for selection of a single gene identifier, the alternative for entering a list, together with the output (expandable by clicking the triangles) from a small example batch query containing one invalid identifier.

Table S1 Available tissue selections for Profile search

Stage	Sex	Tissue	Adult & Larval	Adult only	Male adult	Female adult	Larval only	Alimentary Tract
Adult	Male	Head	Yes	Yes	Yes			
Adult	Female	Head				Yes		
Adult	Male	Eye			Yes			
Adult	Female	Eye		Yes		Yes		
Adult	Male	Brain		Yes	Yes			
Adult	Female	Brain				Yes		
Adult	Male	Thoracicoabdominal ganglion		Yes	Yes			
Adult	Female	Thoracicoabdominal ganglion	Yes			Yes		
Adult	Male	Crop	Yes		Yes			Yes
Adult	Female	Crop		Yes		Yes		Yes
Adult	Male	Midgut		Yes	Yes			Yes
Adult	Female	Midgut	Yes			Yes		Yes
Adult	Male	Hindgut	Yes		Yes			Yes
Adult	Female	Hindgut		Yes		Yes		Yes
Adult	Male	Malpighian Tubules		Yes	Yes			Yes
Adult	Female	Malpighian Tubules	Yes			Yes		Yes
Adult	Male	Fat body	Yes		Yes			
Adult	Female	Fat body		Yes		Yes		
Adult	Male	Salivary gland		Yes	Yes			
Adult	Female	Salivary gland	Yes			Yes		
Adult	Male	Heart	Yes		Yes			
Adult	Female	Heart		Yes		Yes		
Adult	Male	Carcass			Yes			
Adult	Female	Carcass				Yes		
Adult	Male	Rectal pad		Yes	Yes			Yes
Adult	Female	Rectal pad	Yes			Yes		Yes
Adult	Male	Testis	Yes	Yes	Yes			
Adult	Male	Accessory glands	Yes	Yes	Yes			
Adult	Female	Ovary	Yes	Yes		Yes		
Adult	Female	Virgin Spermatheca		Yes		Yes		
Adult	Female	Mated Spermatheca	Yes	Yes		Yes		
Larva	Both	CNS	Yes				Yes	
Larva	Both	Midgut	Yes				Yes	
Larva	Both	Hindgut	Yes				Yes	
Larva	Both	Malpighian Tubules	Yes				Yes	
Larva	Both	Fat body	Yes				Yes	
Larva	Both	Salivary gland	Yes				Yes	
Larva	Both	Trachea	Yes				Yes	
Larva	Both	Carcass					Yes	
Adult	Male	Whole body						
Adult	Female	Whole body						
Larva	Both	Whole body						
Larva	Both	Garland cells					Yes	

The table shows the tissues included in the various alternative profiles offered in the Profile search.

Table S2 FlyBase groups employed in Category search

Group Name	FBgg	Count
Actins	FBgg0000184	6
Acyl-CoA Binding proteins	FBgg0000962	9
Acylglycerol Lipases	FBgg0001376	9
Acyltransferases	FBgg0001168	185
ADAMTS-like	FBgg0001536	3
Aminoacyl-tRNA Synthetases	FBgg0000368	35
Angiotensin-Converting Enzyme-like	FBgg0001388	6
Annexins	FBgg0000164	3
Antimicrobial Peptides	FBgg0001101	23
ATP-Binding Cassette Transporters	FBgg0000547	52
ATPase-coupled Translocases	FBgg0001577	76
Autophagy-related	FBgg0000076	20
Bearded Gene family	FBgg0001065	8
BEAT family	FBgg0000596	14
Bloc1-related Complex	FBgg0001619	8
C-Type Lectin-like	FBgg0001177	39
Cadherins	FBgg0000105	17
Chaperones and Co-Chaperones	FBgg0001643	95
Chemoreceptors	FBgg0000119	121
Chitin-binding	FBgg0001186	106
Chorion proteins	FBgg0001188	9
Chromatin Modifying	FBgg0000300	91
Chromatin Remodeling	FBgg0000284	49
Chromosomal Passenger Complex	FBgg0000127	5
Collagens	FBgg0001185	4
Compass-Type Complexes	FBgg0000313	16
Cop9 Signalosome	FBgg0000152	9
Core Binding Factor	FBgg0000769	6
Cuticle Protein families	FBgg0001178	162
Cytochrome P450	FBgg0001222	87
Deubiquitinases	FBgg0000165	41
DNA Helicases	FBgg0001582	28
DNA Polymerases	FBgg0001200	19
DNA Topoisomerases	FBgg0000806	5
Dorsal Group	FBgg0001054	11
Down Syndrome Cell-Adhesion	FBgg0000722	4
Dynein subunits	FBgg0000364	38
Ecdysteroid Kinase-like	FBgg0001618	54
ELBA Boundary Factor Complex	FBgg0000720	3
Elg1 Complex	FBgg0000599	5
Enhancer of Split Gene	FBgg0000271	12
ER Membrane Protein Complex	FBgg0001289	10
Ferritins	FBgg0000649	3
G protein-coupled Receptors	FBgg0000172	111
Gamma Secretase Complex	FBgg0001062	4
Gemins	FBgg0000470	6
Glue proteins	FBgg0001189	8
Glycoside Hydrolase Family 18	FBgg0000496	17
Glycosyltransferases	FBgg0000795	174
Guanylate Cyclases	FBgg0000515	13
Heat Shock Proteins	FBgg0000501	87
Heterochromatin Pr-1 family	FBgg0000213	5
Histone Lysine Demethylases	FBgg0000307	11
Histone mRNA 3'-processing	FBgg0000630	20
Hox Gene Complex	FBgg0000363	8
Innexins	FBgg0000112	8
Integrins	FBgg0000058	7
Irre Cell Recognition Module	FBgg0001644	4
Kekkons	FBgg0000701	6
Laminins	FBgg0001190	4
Lipid Kinases	FBgg0000639	19
Lysine Deacetylases	FBgg0000290	10
Lysosome-related Biogenesis	FBgg0000163	12
M6A Methyltransferase Complex	FBgg0000702	5
Metallothioneins	FBgg0000197	6
Methyltransferases	FBgg0001159	129
Microtubule Motors	FBgg0001578	39
ML Domain Proteins	FBgg0001653	8
Mucins	FBgg0001180	23
Multicopper Oxidases	FBgg0000654	4
Myosins	FBgg0000166	14
Nonaspanins	FBgg0000531	3
Nuclear Pore	FBgg0000146	29
Nucleases	FBgg0001544	101
Nucleoside Kinases etc.	FBgg0001355	23
Odorant-binding Proteins	FBgg0000175	52
Origin Recognition Complex	FBgg0000510	6
Osiris	FBgg0000612	23
Oxidative Phosphorylation	FBgg0000482	119

Table S2 cont.

P24 Transporters	FBgg0000121	9
Peptidoglycan Recognition proteins	FBgg0000602	13
Peroxins	FBgg0000461	15
PI Glycan Anchor Biosynthesis	FBgg0000617	26
Polyadenylation Factors	FBgg0000618	7
Polycomb	FBgg0000309	15
Proteasome	FBgg0000208	50
Protein Kinases	FBgg0000234	227
Protein Phosphatases	FBgg0000264	96
Pseudokinases	FBgg0000223	25
Receptor Ligands	FBgg0001105	107
RHG Proteins	FBgg0000177	6
Rhomboid-like proteins	FBgg0000955	7
Ribosomal Proteins (cytoplasmic)	FBgg0000141	94
Ribosomal Proteins (mitochondrial)	FBgg0000059	75
RISC Loading Complexes	FBgg0001550	6
RNA Exosome Complex	FBgg0000597	10
RNA Helicases	FBgg0001589	60
RNA Polymerases	FBgg0001646	31
Roundabout	FBgg0000716	3
RZZ Complex	FBgg0000096	3
Scaffold, Coat or Adaptor	FBgg0000056	86
Serpins	FBgg0000103	30
SIDE Family	FBgg0000594	8
Signal Recognition Particle	FBgg0001281	8
SMC Complexes	FBgg0000063	18
SNAREs	FBgg0000194	25
snRNPs	FBgg0000631	76
Spondins	FBgg0001181	5
Synaptotagmins and related	FBgg0000083	10
Tethering Factors	FBgg0000102	54
Tetraspanins	FBgg0000532	38
TOR Complexes	FBgg0000911	5
Transcription Export Complex	FBgg0000603	8
Transferrins	FBgg0000646	3
Transition Zone components	FBgg0000607	10
Translation Factors (cytoplasmic)	FBgg0000571	55
Translation Factors (mitochondrial)	FBgg0000572	9
Troponin Complex	FBgg0001546	7
Tubulins	FBgg0000250	12
Ubiquitin	FBgg0000568	5
Vacuolar ATPase subunits	FBgg0000111	36
Vitelline Membrane proteins	FBgg0001184	6
Water Channel proteins	FBgg0000652	8
Wave Regulatory Complex	FBgg0000492	5
Wirins	FBgg0001381	37
Yellow	FBgg0000086	14

The table lists the 126 FlyBase groups available in the Category search facility and the number of genes from each family in the FlyAtlas 2 database.
